# Long Non-coding RNAs and Their Roles in Non-small-cell Lung Cancer

**DOI:** 10.1016/j.gpb.2016.03.007

**Published:** 2016-07-07

**Authors:** Ming-Ming Wei, Guang-Biao Zhou

**Affiliations:** 1 State Key Laboratory of Membrane Biology, Institute of Zoology, Chinese Academy of Sciences, Beijing 100101, China; 2 University of Chinese Academy of Sciences, Beijing 100049, China

**Keywords:** Long non-coding RNA, Non-small-cell lung cancer, Expression spectrum, Biomarker, Therapeutic resistance

## Abstract

As a leading cause of cancer deaths worldwide, lung cancer is a collection of diseases with diverse etiologies which can be broadly classified into small-cell lung cancer (SCLC) and **non-small-cell lung cancer** (NSCLC). Lung cancer is characterized by genomic and epigenomic alterations; however, mechanisms underlying lung tumorigenesis remain to be elucidated. **Long non-coding RNAs** (lncRNAs) are a group of non-coding RNAs that consist of ⩾200 nucleotides but possess low or no protein-coding potential. Accumulating evidence indicates that abnormal expression of lncRNAs is associated with tumorigenesis of various cancers, including lung cancer, through multiple biological mechanisms involving epigenetic, transcriptional, and post-transcriptional alterations. In this review, we highlight the expression and roles of lncRNAs in NSCLC and discuss their potential clinical applications as diagnostic or prognostic **biomarkers**, as well as therapeutic targets.

## Introduction

Lung cancer is the most common cancer and the leading cause of cancer deaths among men and women worldwide. Among all lung cancer cases, non-small-cell lung cancers (NSCLCs) account for approximately 85% [1], which are at locally advanced or metastatic stage at diagnosis [2]. Based on its pathological characteristics, NSCLC is subdivided into three subtypes, namely, lung adenocarcinoma (LAD), large cell carcinoma (LCC), and lung squamous cell carcinoma (LSCC). LAD and LSCC are the predominant types of NSCLCs, which constitute ∼50% and ∼40% of NSCLC cases, respectively [3]. Although the traditional therapeutic strategies have been tremendously improved and targeted therapies, such as tyrosine kinase inhibitors (TKIs) of the epidermal growth factor receptor (EGFR) [4] and immune checkpoint inhibitors, have been successfully used in clinical practice [5], the five-year overall survival rate of lung cancer of all stages combined remains as low as 15.9% [6]. Such unfavorable outcome could be at least partially attributed to the poor understanding of the pathogenesis of NSCLC, as well as lack of early diagnostic biomarkers and therapeutic targets.

Genetic and epigenetic alterations have been widely recognized as the driving events of cancer. A recent high-throughput transcriptome analysis showed that nearly 75% of the human genome is transcribed into RNAs and only ∼2% of the genome serves as blueprints for proteins with others as non-coding RNAs (ncRNAs) [7]. ncRNAs can be short or small (<200 bp) or long (⩾200 bp) in length. Small ncRNAs include microRNAs (miRNAs), small interfering RNA (siRNAs), PIWI-interacting RNAs (piRNAs), as well as classical housekeeping ncRNAs such as tRNAs, rRNAs, small nuclear RNAs (snRNAs), and small nucleolar RNAs (snoRNAs). miRNAs and piRNAs have been implicated in multiple cellular functions that are essential for physiological or pathological processes [8]. Linearized ncRNAs containing >200 nucleotides are termed as long ncRNAs (lncRNAs), which have attracted much attention recently. A wealth of compelling evidence has demonstrated that aberrantly expressed lncRNAs play important roles in cancer development, including cancer cell proliferation and metastasis, through distinct transcriptional, post-transcriptional, or epigenetic mechanisms [9], [10]. In this review, we focus on the roles of lncRNAs in lung tumorigenesis and briefly introduce the development of lncRNA-directed diagnostics, prognostics and therapeutics.

## Discovery of lncRNAs

The discovery of lncRNAs is attributed to studies on the size, evolution, and function of the genome. Higher species were previously thought to need more genes than lower species [11]. However, developmental complexity of animals is not determined by the amount of DNA in the genome [12]. For example, the genome of salamander is 15 times larger than that of humans [13]. With the aid of DNA–RNA hybridization technique, scientists have come to realize that most parts of the genome do not encode proteins and these non-coding regions of the genome were considered as “junk DNA” [14]. On the other hand, some researchers reckoned that junk DNA was not completely useless [11]. Therefore, substantial interest has been focused on determining the functional roles of these non-coding sequences. As a result, heterogeneous nuclear RNAs and introns were discovered in the 1970s [15], [16], [17]. Subsequent studies demonstrated that snRNAs and snoRNAs play important roles in post-transcriptional RNA processing [18], thus pushing forward investigations on other non-coding sequences. In the early 1990s, the roles of some lncRNAs (*e.g.*, *H19* and *Xist*) in epigenetic regulation were uncovered [19], [20], [21]. However, research on lncRNAs was suspended due to the discovery of miRNAs in 1993 [22] and sustaining keen interests in microRNA studies. Notably, introduction of whole-transcriptome sequencing in the early 2000s led to the identification and annotation of many lncRNAs [23], [24], [25]. A small number of characterized human lncRNAs were then recognized as the central regulators of a variety of biological processes including gene expression, mRNA processing, and protein translation or transport [26]. Up till now, ten thousands of lncRNAs have been identified in different species. However, functional identification of lncRNAs remains a gigantic challenge.

## Characteristics of lncRNAs

**Figure 1:**
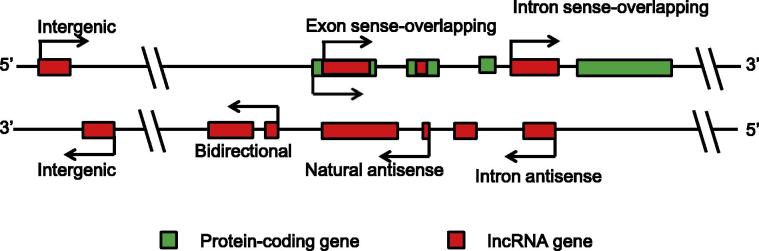
**A diagram of lncRNA categories** Intergenic: a lncRNA gene lies as an independent unit within the genomic interval between two genes. Bidirectional: expression of a lncRNA gene and its neighboring coding transcript on the opposite strand is initiated in close genomic proximity. Intron sense-overlapping: a lncRNA gene lies in the intron of a protein-coding gene on the same strand. Exon sense-overlapping: a lncRNA gene lies in the exons of protein-coding gene on the same strand. Intronic-antisense: a lncRNA lies in the introns of protein-coding gene on the opposite strand in the same region. Natural-antisense: a lncRNA gene lies in the exons of protein-coding gene on the opposite strand. lncRNA, long non-coding RNA.

lncRNAs contain ⩾200 nucleotides and bear no or low translational potential [9]. Based on their relationships with protein-coding genes, lncRNAs are classified into six broad categories, namely, intergenic, bidirectional, intron sense-overlapping, exon sense-overlapping, intronic-antisense, and natural-antisense lncRNAs [27] (Figure 1). lncRNAs usually are transcribed by RNA polymerase II (RNAPII), but there are some exceptions. For instance, brain-associated *BC200* is transcribed by RNAPIII [28]. Generally, lncRNAs are expressed at lower levels and are less conserved than protein-coding genes [29], [30], [31] and some lncRNAs exhibit cell-, tissue- and time-specific expression patterns [32]. A growing body of evidence has indicated that the expression of lncRNAs is tightly regulated through distinct mechanisms, such as chromatin state, transcription factors (TFs), and microRNAs [33]. And majority of lncRNAs are transcribed from antisense regions upstream of promoters, intragenic regions, intergenic regions distal to promoters, or gene bodies of protein-coding genes [7].

## Functions of lncRNAs

**Figure 2:**
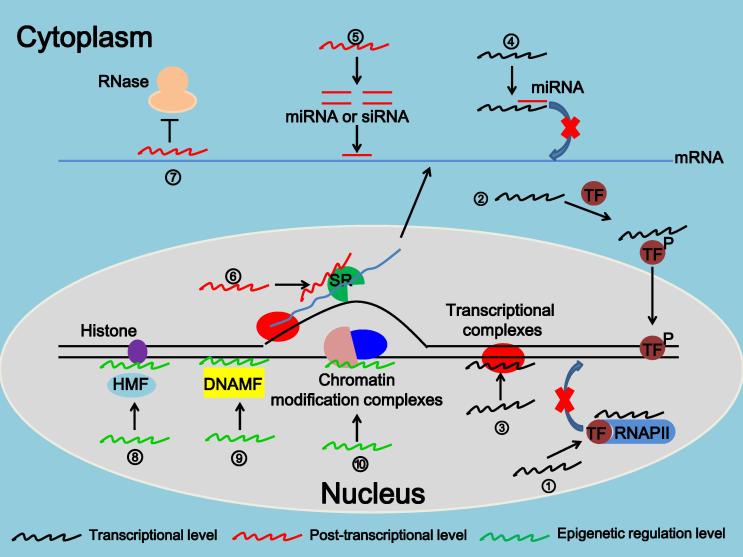
**Molecular mechanisms for the functions of lncRNAs** ① lncRNA acts as decoys for TFs or RNAPII; ② lncRNA alters the modification and location of transcription factors; ③ lncRNA interacts with DNA and forms triple helix structures, thereby recruiting transcriptional complex; ④ lncRNA acts as decoy for miRNA; ⑤ lncRNA acts as precursor for siRNAs or miRNAs; ⑥ lncRNA regulates the alternative splicing of pre-mRNAs through SR complex; ⑦ lncRNA protects mRNA from degradation through forming double-stranded RNA with mRNAs; ⑧ lncRNA regulates histone modification by interacting with modification factors; ⑨ lncRNA binds to DNA modification factors to modify the methylation of DNA; ⑩ lncRNA binds to chromatin modification complexes to regulate chromatin remodeling and structure. DNAMF: DNA modification factor; HMF: histone modification factor; miRNA, microRNA; siRNA, small-interfering RNA; TF, transcription factor; RNAPII, RNA polymerase II.

LncRNAs function in diverse biological processes by modulating the transcription and translation of protein-coding genes. Unlike miRNAs, which commonly participate in mRNA degradation or regulate mRNA translation [34], [35], [36], lncRNAs regulate the expression of target genes through multiple mechanisms at different levels (Figure 2). lncRNAs can interact directly with DNA, mRNA, or proteins to regulate chromatin modification or structure, transcription, splicing, and translation, so as to regulate a variety of physiological and pathological processes such as cell proliferation or differentiation, stem cell reprogramming, tumorigenesis, or drug resistance [10], [37], [38]. Functions of lncRNAs are summarized in Figure 2 and briefly described below.

First, at the transcriptional level, lncRNAs (i) act as decoys for TFs or RNAPII to disrupt their binding to promoters/enhancers of target genes, thus promoting or suppressing gene expression [39]; (ii) interact directly with TFs and alter their modification or localization to regulate gene transcription [40]; (iii) interact with DNA and form scaffolds for TFs, thus affecting target gene transcription [41]; and (iv) act as competitive endogenous RNAs (ceRNAs) to control target gene transcription [42].

Second, at the post-transcriptional level, lncRNAs (i) act as precursors of siRNAs or miRNAs, leading to decreased expression of their target genes [43], (ii) form double-stranded RNA complexes with mRNAs and protect them from degradation [44], and (iii) regulate the alternative splicing of pre-mRNAs to produce different transcripts [45].

Lastly, at the epigenetic level, lncRNAs (i) interact with proteins associated with histone modifications to modify the methylation, acetylation or ubiquitination of histones [46]; (ii) get involved in gene silencing by regulating DNA methylation in the promoter region of target genes [47]; and (iii) get involved in chromatin remodeling or conformational alterations by binding to chromatin modification complexes, which is important for gene transcription [7].

## Expression spectrum of lncRNAs in NSCLCs

Compelling evidence has demonstrated the important roles of lncRNAs in various diseases, particularly in cancer. Recent studies have reported lncRNA expression in NSCLCs. For instance, using high-throughput microarrays, Xu et al identified 2420 lncRNAs that were differentially expressed (fold change ⩾2) between LAD and normal tissue (NT) samples. Of these 2420 lncRNAs, the expression of 1213 lncRNAs was upregulated, whereas the expression of the remaining 1207 lncRNAs was downregulated [48]. As another example, Yang et al identified 47 lncRNAs (14 upregulated and 33 downregulated lncRNAs) from gene expression data of five NSCLC cohorts that were deposited in the Gene Expression Omnibus (GEO) database [49]. Interestingly, several novel lncRNAs were identified to be induced by established risk factors for NSCLC, such as cigarette smoking or exposure to a polycyclic aromatic hydrocarbon compound benzo(a)pyrene (BaP). These include cancer-associated lncRNA-1 (*SCAL1*), *DQ786227*, and *LOC728228* [50], [51], [52]. We recently reported the screening for lncRNAs with abnormal expression in lung cancers that are associated with air pollution [53]. We found that the cancer samples of patients from high pollution region had much more dysregulated lncRNAs than patients from control regions when compared to their corresponding neighboring tissues. Among these, the expression of an lncRNA, *CAR intergenic 10* (*CAR10*), was up-regulated in air pollution-related NSCLCs. Expression of *CAR10* could be upregulated by the carcinogen dibenz[a,h]anthracene (DBA) through increasing expression of TF FoxF2. *CAR10* binds to and stabilizes TF Y-box-binding protein 1 (YB-1), leading to up-regulation of EGFR and proliferation of lung cancer cells. Knockdown of *CAR10* inhibited cell growth *in vitro* and *in vivo*, suggesting the role of lncRNAs in environmental lung carcinogenesis [53]. To gain new insights into the pathogenesis of NSCLCs, the molecular mechanisms underlying the roles of several lncRNAs such as *MALAT1*, *HOTAIR*, *H19*, and *PVT1* have been extensively investigated. We list the majority of known NSCLC-associated lncRNAs and their functions in Table 1. Their potential application as early diagnostic or prognostic biomarkers and efficient therapeutic targets in patients with NSCLCs warrants further investigations.

## lncRNAs as biomarkers of NSCLCs

To improve overall survivals of patients, it is important to exploit new biomarkers for diagnosing, subtyping, and prognosing of NSCLCs. More and more studies have been focused on ncRNAs, particularly miRNAs in the past few years [83]. Likewise, studies have indicated that aberrant expression of lncRNAs is also a hallmark of carcinomas and some lncRNAs show tissue- or cell-specific expression pattern [84], suggesting their potentials as biomarkers. Several lncRNAs have been reported as candidate biomarkers, *e.g.*, highly up-regulated in liver cancer (*HULC*) in human hepatocellular carcinoma [85] and prostate cancer gene 3 (*PCA3*) in prostate cancer [86]. Notably, many dysregulated lncRNAs have been identified in patients with NSCLCs (Table 1), suggesting that lncRNAs could be used for screening effective and specific biomarkers of NSCLCs.

To screen for lncRNAs as biomarkers for LADs at early-stage, Li et al summarized microarray data of 181 patients with early-stage LADs to examine their lncRNA expression profiles. As a result, they found that *LINC00313* was highly expressed in patients with T2- and N1-stage LADs [87]. Therefore, *LINC00313* could be used as a diagnostic biomarker of early-stage LADs. lncRNAs can be detected in serum, which makes it easier for clinical applications. Hence, researchers put more emphasis on circulating lncRNAs. As a results, *MALAT1* [88], *XIST*, and *HIF1A-AS1* [89] were found overexpressed in NSCLC patients’ serum when compared with controls. These lncRNAs may act as diagnosis biomarkers for screening NSCLCs via peripheral blood detection.

Subtyping of NSCLC cases is important for the selection of clinical treatment options. For instance, patients with LAD and LSCC differ in clinical outcomes. Zhao et al identified 72 differentially-expressed (23 upregulated and 49 downregulated) lncRNAs in patients with LADs and LSCCs by using human Affymetrix microarrays (HGU133plus2.0) [90]. Likewise, White et al identified 27 lung cancer-associated lncRNAs, which could be used as novel biomarkers for stratifying LADs and LSCCs [91]. Zhang et al found that expression of a novel lncRNA, *LINC01133*, was upregulated in LSCC but not in LAD samples [92]. All these findings indicate that some lncRNAs could serve as potential biomarkers for distinguishing subgroup of NSCLCs.

lncRNAs could also be used as prognostic biomarkers in patients with NSCLCs. For instance, expression levels of lncRNAs *RP11-21L23.2*, *GPR158-AS1*, *RP11-701P16.5*, and *RP11-379F4.4* were negatively correlated with NSCLC patients’ overall survival. Conversely, expression levels of lncRNAs *CTD-2358C21.4*, *RP11-94L15.2*, *KCNK15-AS1*, and *AC104134.2* were positively associated with the overall survival of NSCLC patients [93].

The observations above indicate that despite their obscure roles in lung tumorigenesis, these lncRNAs may be valuable for diagnosis of NSCLCs, selecting treatment protocols, and predicting the prognosis of patients with NSCLCs.

## lncRNAs in the therapeutic resistance of NSCLCs

At present, surgical excision, chemotherapy, chest radiotherapy and targeted therapy are used alone or in combination to treat patients with NSCLC [94]. However, drug therapies fail in most NSCLCs due to development of drug resistance [95]. Studies have suggested an important role of dysregulated miRNAs in the development of drug resistance [96]. Additional studies also have demonstrated the association between the expression of certain lncRNAs and chemotherapeutic sensitivity of cancer cells. For instance, *H19* induced P-glycoprotein- and MDR1-associated drug resistance in liver cancer cells [97].

Resistance to cisplatin, carboplatin, and EGFR-TKIs is inevitable in treating NSCLCs [98]. In an effort to explore the molecular mechanisms of cisplatin resistance, Yang et al found 1380 lncRNAs differentially expressed between regular A549 and cisplatin-resistant A549 cells, indicating the possible involvement of lncRNAs in cisplatin resistance. The authors identified a novel lncRNA, *AK126698*, which confers cisplatin resistance by targeting the Wnt pathway [82]. Likewise, other research groups showed that *HOTAIR* contributed to cisplatin resistance of NSCLC cells by downregulating p21^WAF1/CIP1^ expression [99] and that *MEG3* mediated cisplatin resistance of NSCLC cells by regulating the expression of p53 and Bcl-xL [78]. Patients with low *MEG3* expression showed poor response to cisplatin-based chemotherapy [78]. Notably, the effectiveness of cisplatin against LSCCs varied between individuals due to different gene expression profiles [100]. For instance, cisplatin-based chemotherapy was beneficial for LSCC patients with excision repair cross-complementation group 1 (ERCC1)-negative tumors after surgical operation, but not for LSCC patients with ERCC1-positive tumors [101]. Hou et al identified 1702 lncRNAs that were differentially expressed between cisplatin-sensitive and cisplatin-resistant LSCC patients. In particular, the expression of *AC006050.3-003* was significantly downregulated in patients showing sensitivity to cisplatin compared with those with resistance to cisplatin, suggesting that *AC006050.3-003* may be a biomarker for cisplatin treatment in patients with LSCCs [102]. In addition, Dong et al found that *GAS5* enhanced the sensitivity of cells expressing wild-type EGFR to gefitinib treatment [103]. These studies demonstrate the correlations between lncRNAs and drug resistance, providing additional opportunities to overcome drug resistance by targeting lncRNAs and related signaling pathways.

## Conclusions and perspectives

With the development of technological approaches, such as lncRNA microarray and RNA sequencing, more and more lncRNAs have been found to be dysregulated in NSCLCs, which function as oncogenes or tumor suppressors. Some of these lncRNAs are associated with different stages of NSCLCs, some are specifically overexpressed in one of the lung cancer subtypes, and some are involved in drug resistance. These findings suggest the important roles of lncRNAs in the pathogenesis and treatment of NSCLCs. However, only a small number of lncRNAs have been well characterized, whereas functions of most lncRNAs remain to be elucidated.

Many key questions still need to be addressed. For example, how lncRNAs regulate downstream pathways? Can we use lncRNAs as predictive markers for lung cancer risk or as early diagnostic or prognostic markers? How do lncRNAs mediate drug resistance? Can we use lncRNAs as appropriate therapeutic targets, how to target them if yes? How do we deliver the therapeutic lncRNAs into target tissues and evaluate their safety? Answers to these and other questions will provide new insights into the pathogenesis of lung cancers and help optimize therapeutic strategies to improve the clinical outcome of this deadly disease, which causes 1.59 million deaths each year worldwide [104].

## Competing interests

The authors have declared that there are no competing interests.

## Figures and Tables

**Table 1:** NSCLC-associated lncRNAs

**lncRNA**	**Expression**	**Key factors**	**Functions**	**Ref.**
*CAR10*	Up	YB-1	Promote cell proliferation	[53]
*MALAT1*	Up	SR, PC2, hnRNP C	Promote cell proliferation, migration, and invasion	[54]
*HOTAIR*	Up	PRC2, LSD1	Promote cell proliferation, invasion, and metastasis	[55]
*H19*	Up	miR-675, c-MYC, p53	Suppress apoptosis Promote cell growth	[56]
*RGMBAS1*	Up	RGMB	Promote cell metastasis	[57]
*PVT1*	Up		Promote cell proliferation, migration, and invasion	[58]
*GHSROS*	Up		Promote cell migration	[59]
*NKX2-AS1*	Up	EZH2, UTX	Promote cell growth, Regulate cell shape	[60]
*BCYRN1*	Up	c-MYC	Promote cell motility, migration, and invasion	[61]
*DLX6-AS1*	Up	DLX6	Carcinogenesis	[62]
*AFAP1-AS1*	Up	Actin filament integrity	Promote cancer cell metastasis	[63]
*SOX2-OT*	Up	PRC2	Promote cell proliferation	[64]
*CARLo-5*	Up		Promote cell proliferation, migration, and invasion,	[65]
*Lnc_bc060912*	Up	PARP1, NPM1	Repress cell apoptosis	[66]
*MVIH*	Up		Promote cell proliferation and invasion	[67]
*HNF1A-AS1*	Up	DNMT1	Promote tumor proliferation and metastasis	[68]
*CCAT2*	Up		Promote cell proliferation and invasion	[69]
*LUADT1*	Up	SUZ12, p27, LUAD	Regulate cell cycle	[70]
*ZXF1*	Up		Promote cell invasion and metastasis	[71]
*ANRIL*	Up	PRC2	Correlate with TNM stages and tumor size	[72]
*SCAL1*	Up	Nrf-2	Mediate oxidative stress protection	[52]
*NRG1*	Up		Carcinogenesis	[73]
*DQ786227*	Up		Mediate oxidative stress protection	[50]
*LOC728228*	Up		Mediate oxidative stress protection	[51]
*GAS5*	Down	p53, E2F1, miR-21	Induce apoptosis, drug resistance	[74]
*GAS6-AS1*	Down		Suppress metastasis	[75]
*PANDAR*	Down	p53, NF-YA, Bcl-2	Repress cell proliferation	[76]
*HMlincRNA717*	Down		Associate with lymph node metastasis	[77]
*MEG3*	Down	P53	Suppress cell proliferation, Induce apoptosis	[78]
*TUG1*	Down	P53, PRC2	Suppress cell proliferation	[79]
*SPRY4-IT1*	Down	PRC2	Induce apoptosis, Suppress cell proliferation	[80]
*BANCR*	Down		Suppress cell proliferation, Induce apoptosis	[81]
*AK126698*	Down	Wnt pathway	Mediate cisplatin resistance	[82]

*Note:* AFAP1-AS1, actin filament associated protein 1 antisense RNA 1; ANRIL, antisense noncoding RNA in the INK4 locus; BANCR, BRAF-activated non-coding RNA; BCYRN1, brain cytoplasmic RNA 1; CAR10, chromatin associated RNA intergenic 10; CARLo-5, also known as colon cancer associated transcript 1 (CCAT1); CCAT2, colon cancer associated transcript 2; DLX6, distal-less homeobox 6; DLX6-AS1, distal-less homeobox 6 antisense RNA 1; DNMT1, DNA methyltransferase 1; EZH2, enhancer of Zeste homolog 2; GAS5, growth arrest-specific transcript 5; GAS6-AS1, growth arrest-specific transcript 6 antisense RNA 1; GHSROS, growth hormone secretagogue receptor opposite strand; HNF1A-AS1, HNF1 homeobox A antisense RNA 1; hnRNP C, heterogeneous nuclear ribonucleoprotein C; HOTAIR: Hox antisense intergenic RNA; lncRNA, long non-coding RNA; LSD1, lysine-specific demethylase 1; LUADT1, lung adenocarcinoma associated transcript 1; MALAT1, metastasis associated lung adenocarcinoma transcript 1; MEG3, maternally expressed gene 3; MVIH, microvascular invasion in HCC; NF-YA, A subunit of nuclear factor-Y; NKX2-AS1, NK2 homeobox-1 antisense RNA 1; NPM1, nucleophosmin 1; Nrf-2, NF-E2-related factor 2; NRG1, nickel-related gene 1; NSCLC, non-small-cell lung cancer; PANDAR, promoter of CDKN1A antisense DNA damage activated RNA; PARP1, poly (ADP-ribose) polymerase 1; PC2, subtilisin-related proprotein convertases 2; PRC2, polycomb repressive complex 2; PVT1, plasmacytoma variant translocation 1; RGMB, repulsive guidance molecule b; RGMBAS1, repulsive guidance molecule b antisense RNA1; SCAL1, smoke and cancer-associated lncRNA-1; SOX2-OT, SRY-box 2 overlapping transcript; SPRY4-IT1, SPRY4 intronic transcript 1; SR, serine/arginine RNA splicing protein; SUZ12, suppressor of Zeste 12; TUG1, taurine-upregulated gene 1; UTX, lysine demethylase 6A; YB-1, Y-box-binding protein 1; ZXF1, as known as ACTA2 antisense RNA 1 (ACTA2-AS1).
